# The diagnostic dilemma of adrenal vascular tumors: analysis of 21 cases and systematic review of the literature

**DOI:** 10.1007/s12020-024-04123-5

**Published:** 2025-01-18

**Authors:** Kimberly Coscia, Caterina Ravaioli, Lorenzo Tucci, Giacomo Colombin, Francesca Donnarumma, Cristina Mosconi, Caterina Balacchi, Cristina Nanni, Laura Alberici, Saverio Selva, Uberto Pagotto, Donatella Santini, Giovanni Tallini, Guido Di Dalmazi, Valentina Vicennati, Antonio De Leo

**Affiliations:** 1 Division of Endocrinology and Diabetes Prevention and Care, IRCCS Azienda Ospedaliero-Universitaria di Bologna, Bologna, Italy; 2 Department of Medical and Surgical Sciences (DIMEC), Alma Mater Studiorum University of Bologna, Bologna, Italy; 3 Anatomic Pathology - Department of Medical and Surgical Sciences (DIMEC), University of Bologna, Bologna, Italy; 4 Solid Tumor Molecular Pathology Laboratory, IRCCS Azienda Ospedaliero-Universitaria di Bologna, Bologna, Italy; 5 Department of Specialized, Radiology Unit, Diagnostic and Experimental Medicine (DIMES), Alma Mater Studiorum University of Bologna, IRCCS Azienda Ospedaliero-Universitaria di Bologna, Bologna, Italy; 6 Nuclear Medicine, IRCCS Azienda Ospedaliero-Universitaria di Bologna, Bologna, Italy; 7 Division of Pancreatic and Endocrine Surgical Unit, IRCCS Azienda Ospedaliero-Universitaria di Bologna, Bologna, Italy

**Keywords:** Adrenal tumors, Adrenal endothelial cysts, Adrenal hemangiomas, Adrenal lymphangiomas.

## Abstract

**Purpose:**

Adrenal vascular tumors are mainly represented by adrenal cavernous hemangiomas (ACHs) and adrenal cystic lymphangiomas (ACLs). Their radiological features often overlap with malignant tumors, therefore ruling out malignancy becomes mandatory. We analyzed clinical, radiological, and histopathological data to identify specific characteristics of these tumors.

**Methods:**

We reviewed 21 patients with ACHs (n = 12), ACLs (n = 8), or adrenal cysts (n = 1) confirmed by histopathology. We selected 82 papers from PubMed to provide a systematic review of the literature.

**Results:**

In our cohort, median age at diagnosis was 58 years, with sex evenly distributed. All tumors were unilateral (median size = 44 mm), with 6 cases of increasing tumor size. All tumors exhibited non-contrast CT density > 10 Hounsfield Unit (HU). Calcifications were found in 5 cases. Hormonal studies revealed 11 non-functioning tumors and 2 cortisol-secreting tumors. Elevated urinary metanephrines were found in 2 cases. Immunostaining showed CD31/CD34/factor VIII expression in ACHs (n = 5, 24%) and podoplanin expression in ACLs (n = 6, 29%). The literature review revealed 71 reported cases of ACHs and 104 reported cases of ACLs. Median age at diagnosis was 46 years, with slightly female prevalence (63%). Median tumor size was 48 mm. 84 cases were symptomatic, with life-threatening hemorrhage reported in only 3 patients. Calcifications were found in 23% of cases. Surgical approaches varied, with open and laparoscopic adrenalectomy performed in 55 and 42 patients respectively.

**Conclusions:**

ACHs and ACLs represent a diagnostic dilemma in clinical practice due to their rarity and their misleading imaging features.

## Introduction

Adrenal cysts (ACs) are considered rare radiological findings, comprising approximately 1–2% of all adrenal incidentalomas, with an overall incidence ranging between 0.064% and 0.18% [1, 2]. They are subdivided into pseudocysts, endothelial cysts, epithelial cysts, and parasitic cysts. Adrenal vascular cysts account for 20–32% of all ACs. They can be classified into angiomatous or lymphatic according to the histological origin of the endothelium [3, 4]. They are mainly represented by adrenal cavernous hemangiomas (ACHs) and adrenal cystic lymphangiomas (ACLs) [1]. Both ACHs and ACLs are usually unilateral, benign, non-functioning, and asymptomatic, but differential diagnosis with other adrenal masses before surgical removal may be challenging. Symptoms, such as dull abdominal or flank pain, may occur if tumor size increases, thus determining mass effect on adjacent structures [1]. In addition, the risk of life-threatening retroperitoneal hemorrhage is proportionally related to tumor size. Radiological characteristics often overlap with pheochromocytomas and adrenal carcinomas, therefore, ruling out malignancy becomes mandatory. Diagnosis is usually made on histopathology, whereas pre-operative diagnosis remains a critical issue in clinical practice [1]. We analyzed clinical, radiological, and histopathological data to identify characteristics and outcomes of adrenal vascular tumors. A review of the literature is also provided.

## Material and methods

**Fig. 1:**
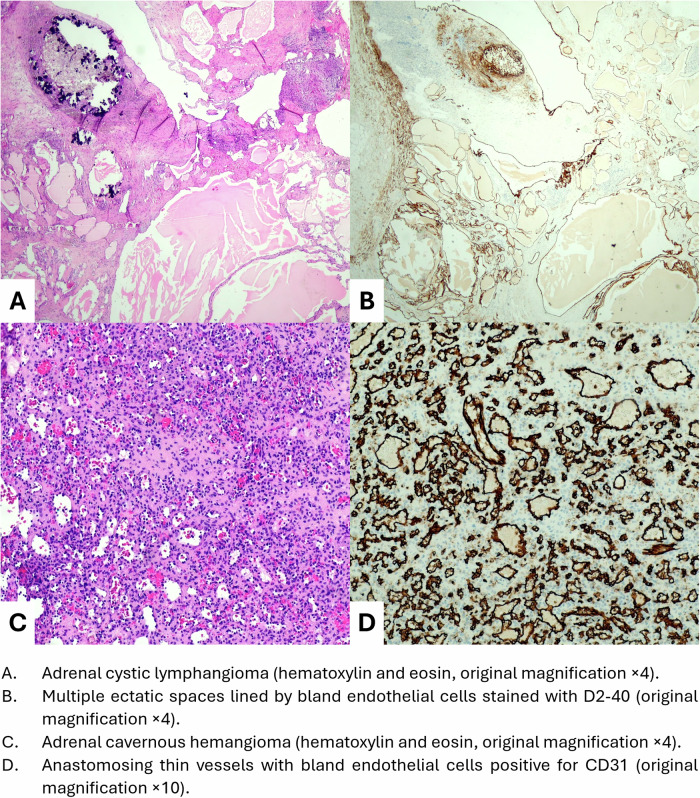
Photographs of adrenal cystic lymphangioma (**A**, **B**) and adrenal cavernous hemangioma (**C**, **D**) on histopathology. **A** Adrenal cystic lymphangioma (hematoxylin and eosin, original magnification ×4). **B** Multiple ectatic spaces lined by bland endothelial cells stained with D2-40 (original magnification ×4). **C** Adrenal cavernous hemangioma (hematoxylin and eosin, original magnification ×4). **D** Anastomosing thin vessels with bland endothelial cells positive for CD31 (original magnification ×10)

We retrospectively enrolled 21 consecutive patients admitted to S. Orsola IRCCS Polyclinic of Bologna between 2007 and 2022, who received a diagnosis of ACH or ACL confirmed by histopathology (Fig. 1). One of these patients was previously presented as a case report [5]. Descriptive data, including demographics, tumor size, clinical presentation, hormonal assessment, adrenal imaging features, surgical treatment, histopathology, and clinical outcomes were collected retrospectively from electronic health records. Hormonal evaluation was available in 15 patients. In the presence of atypical adrenal tumors (non-contrast computerized tomography [CT] density > 10 Hounsfield Units [HU]), urinary and/or plasma metanephrines and androgens were assessed (n = 15, 71%). In patients with concomitant hypertension (n = 6, 29%), plasma aldosterone to renin ratio was evaluated as well, according to primary hyperaldosteronism guidelines and after discontinuing interfering medications [6]. In all cases, 1 mg-dexamethasone suppression test (DST) was performed to identify mild autonomous cortisol secretion (MACS). Adrenal tumors were defined as non-secreting for post-DST serum cortisol values ≤ 1.8 mcg/dL (50 nmol/L), whereas MACS was detected for post-DST serum cortisol values above this cut-off. All CT scans were reviewed by two expert radiologists (CM and CB) to collect dimensions and measurements of unenhanced density using region-of-interest calculations, expressed as HU.

Atypical adrenal tumors were characterized by non-contrast CT density > 10 HU. Adrenal lipid-poor adenomas were further defined by analyzing absolute and relative washout values from non-enhanced, portal venous phase, and 15-minute delayed CT scans. Thresholds were established at 60% and 40%, respectively, according to previous adrenal incidentalomas guidelines [7]. For adrenal vascular cysts, contrast-enhanced CT scans were used to assess wall thickness, peripheral contrast enhancement, septations, calcifications, solid components, and hemorrhage. Increasing trend in tumor size was evaluated during the presurgical follow-up. We considered significant tumor growth according to the RECIST 1.1 criteria [8]. Magnetic resonance imaging (MRI) was performed in 7 patients as additional imaging. ACHs showed peripheral spotty enhancement and hyperdense peripheral rim, while ACLs were identified as lobulated and septate lesions with fluid attenuation values (<20 HU) and thin enhancing walls. All histopathological slides were reviewed by two expert pathologists (ADL and DS). Immunohistochemical analysis was performed on 11 out of 21 cases where morphological evaluation alone was insufficient to confirm the vascular nature of the cysts. The remaining cases were assessed with hematoxylin and eosin staining, as their morphology was consistent with ACHs or ACLs. The antigen panel included CD31, CD34, factor VIII, and podoplanin, typically positive in adrenal vascular cysts. Calretinin was also included to exclude epithelial cysts. Data are presented as descriptive statistics.

Furthermore, we conducted an extensive literature search on PubMed using the keywords “adrenal hemangiomas” and “adrenal lymphangiomas”. Original articles, case reports, and systematic reviews published between 1950 and 2022 were considered. Only articles in English were included. We initially screened 440 papers based on their titles and abstracts. Following a full-text review, we subsequently selected 106 papers, with 82 papers considered for the systematic literature analysis. We overall reviewed 71 reported cases of ACHs and 104 reported cases of ACLs.

## Results

### Our cohort

Descriptive data of the study population was reported in Table 1. We enrolled 21 patients with ACHs (n = 12; 57%), ACLs (n = 8; 38%), or adrenal cysts (n = 1; 5%) confirmed by histopathology. More than half of the cases were incidentally discovered (n = 14; 67%) and only 3 cases were identified correctly by pre-operative imaging studies. Median age at the diagnosis was 58 years (range 30–75 years). Sex was evenly distributed (females n = 9, 43%; males n = 12, 57%). All tumors were unilateral. Median preoperative tumor size was 44 mm (range 9–95 mm), with similar median tumor size in the pathological report (40 mm; range 7–150 mm). A significant upward trend in tumor size during the pre-surgical follow-up (mean value = 4 years) was observed in 6 cases. All tumors showed CT density values on the non-contrast series > 10 HU (median = 29 HU, range 11–44 HU). Calcifications were found in 5 patients. Elevated urinary metanephrines were found in 2 cases. Hormonal studies revealed non-functioning adrenal masses in 11 cases and MACS in 2 patients (post-DST serum cortisol values ≤ 1.8 mcg/dL in 13 cases; post-DST serum cortisol values > 1.8 mcg/dL in 2 cases). None of the cases showed impairments in the mineralocorticoid and androgens secretion. Clinical, radiological, and biochemical preoperative studies raised the suspicion of pheochromocytoma (n = 4; 19%), atypical adrenal tumor (n = 5; 24%), adrenal lipid-poor adenoma (n = 1; 5%), adrenal hemangioma (n = 1; 5%), adrenal cystic lymphangioma (n = 2; 10%), and adrenal cyst (n = 2; 10%). Most patients were asymptomatic, as only 4 patients presented with non-specific symptoms (abdominal pain). Laparoscopic adrenalectomy was performed in all patients, with only one case of conversion to open adrenalectomy due to intraoperative bleeding. Immunohistochemical analysis was performed in 11 out of 21 cases. ACHs (n = 524%) demonstrated CD31 and/or CD34 and/or factor VIII expression, while ACLs (n = 6, 29%) were found to be positive for podoplanin.

**Table 1:** Descriptive data of our cohort (N = 21)

Characteristics	N° (%)	Median	Range (min-max)
Age (years)	21 (100)	58	30–75
Females	9 (43)		
Males	12 (57)		
Incidental finding	14 (67)		
Right site	9 (43)		
Left site	12 (57)		
Mean tumor size (mm)	15 (71)	44	9–95
Increased tumor size	6 (29)		
Symptomatic	4 (19)		
Hormonal secretion	4 (19)		
*Imaging features*			
CT density on the non-contrast series (HU)	15 (71)	29	11–44
CT density of portal venous phase (HU)	14 (67)	72	14–171
CT density of 15 minutes delayed CT scans (HU)	14 (67)	73	23–88
Calcifications	5 (24)		
*Pre-operative diagnosis*			
Pheochromocytoma	4 (19)		
Atypical adrenal tumor	5 (24)		
Adrenal lipid-poor adenoma	1 (5)		
Adrenal cystic lymphangioma	2 (10)		
Adrenal hemangioma	1 (5)		
Adrenal cyst	2 (10)		
*Diagnosis on histopathology*			
Adrenal hemangioma	12 (57)		
Adrenal cystic lymphangioma	8 (38)		
Adrenal cyst	1 (5)		

*CT* Computerized Tomography, *HU* Houndsfield Units.

### Systematic review of the literature

After an extensive literature review, comprising an initial screening of 440 papers, we identified 82 papers reporting a total of 71 cases of ACHs and 104 cases of ACLs (median number of patients for each study = 1, range 1–37). Data of the reported cases was provided in Table 2. Median age at diagnosis was 46 years (range 5–84 years). Most of the patients were female (n = 111; 63%). Median tumor size at diagnosis was 48 mm (range 15–350 mm). All but two cases of ACHs and ACLs were unilateral. About half of the cases were detected as asymptomatic incidentalomas (n = 91; 52%). Among symptomatic cases (n = 84; 48%), the most common symptom was abdominal pain (n = 68; 81%), followed by hypertension (n = 13; 15%). Only 3 cases (4%) presented with life-threatening retroperitoneal hemorrhage. Functional studies showed 12% of patients with hormonal hypersecretion (catecholamines or metanephrines elevation n = 10, 6%; MACS n = 4, 2%; primary hyperaldosteronism n = 2, 1%; Cushing’s syndrome n = 1; 1%; cortisol and aldosterone co-secretion n = 3; 2%). CT scans were performed in 100 patients (57%), with 16 tumors showing a high density in the non-contrast phase (9%). However, most studies did not provide information about density values on CT scans. Calcifications were described in 41 patients (23%). About one-third of the patients underwent open adrenalectomy (n = 55; 31%). Only 23% of the cases underwent laparoscopic adrenalectomy (n = 40), whereas the surgical technique was not specified in the remaining cases (n = 80; 46%).

**Table 2:** ACHs and ACLs reported cases in the literature (N = 175) and our cohort (N = 21)

Authors/year	N° of cases	Sex/age	Site	Size (mm)	Symptoms & Signs	Hormonal secretion	Imaging at diagnosis	Diagnosis on histopathology	Intraperitoneal emorrhage	Treatment
Johnson/1955 [10]	1	F/46 yy	Right	N/A	No	N/A	US	Adrenal cavernous hemangioma	No	Open adrenalectomy
Chodoff/1966 [11]	1	F/76 yy	Left	160	Yes	N/A	N/A	Adrenal cavernous hemangioma	No	Open adrenalectomy
Weiss/1966 [37]	1	M/70 yy	Right	110	No	No	N/A	Adrenal hemangioma	No	Open adrenalectomy
Vargas/1980 [38]	1	F/67 yy	Left	140	No	No	US	Adrenal cavernous hemangioma	No	Open adrenalectomy
Lee/1982 [39]	1	F/59 yy	Right	85	Yes	No	CT	Adrenal hemangioma	No	Open adrenalectomy
Goren/1986 [40]	1	F/79 yy	Right	60	No	No	CT	Adrenal cavernous hemangioma	No	Open adrenalectomy
Derchi/1989 [41]	2	F = 1 M = 1 65 yy	Right=1	190	Yes = 1 No = 1	No	US = 2, CT = 2	Adrenal cavernous hemangioma	No	Open adrenalectomy
Salup/1992 [16]	1	F/74 yy	Left	150	No	No	CT	Adrenal cavernous hemangioma	No	Open adrenalectomy
Deckers/1993 [42]	1	F/56 yy	Right	65	No	No	US, CT	Adrenal cavernous hemangioma	No	Open adrenalectomy
Hamrick-Turner/1994 [34]	1	M/66 yy	Left	140	Yes	No	CT	Adrenal cavernous hemangioma	No	Open adrenalectomy
Boraschi/1995 [43]	1	M/64 yy	Right	80	Yes	No	CT	Adrenal cavernous hemangioma	No	Open adrenalectomy
Ness/1996 [44]	1	M/35 yy	Right	80	Yes	No	CT, MRI	Adrenal cystic lymphangioma	No	Open adrenalectomy
Stumvoll/1996 [21]	1	F/60 yy	Right	80	No	Yes	CT	Adrenal cavernous hemangioma	No	Open adrenalectomy
Oh/1997 [45]	1	M/56 yy	Right	60	Yes	No	US, CT, MRI	Adrenal cavernous hemangioma	No	Open adrenalectomy
Milosrdnice/1997 [46]	2	F = 1 M = 1 64 yy	Left	110	Yes = 1 No = 1	No	US = 2, CT = 2	Adrenal cavernous hemangioma	No	Open adrenalectomy
Hoeffel/1999 [27]	1	F/22 yy	Bilateral	78	Yes	No	CT	Adrenal cystic lymphangioma	No	Open adrenalectomy
Longo/2000 [47]	1	F/30 yy	Right	48	Yes	No	CT, MRI	Adrenal cystic lymphangioma	No	Open adrenalectomy
Thiele/2001 [48]	1	F/72 yy	Left	60	No	No	CT	Adrenal hemangioma	No	Open adrenalectomy
Hui-Xiong/2003 [49]	1	M/66 yy	Right	150	No	No	US, CT, MRI	Adrenal cavernous hemangioma	No	Open adrenalectomy
Satou/2003 [50]	1	M/46 yy	Left	30	No	No	CT, MRI	Adrenal cystic lymphangioma	No	Laparoscopic adrenalectomy
Garcia/2004 [51]	1	F/22 yy	Left	40	Yes	No	CT	Adrenal cystic lymphangioma	No	Open adrenalectomy
Ates/2005 [52]	1	F/ 26 yy	Right	70	Yes	No	CT, MRI	Adrenal cystic lymphangioma	No	Open adrenalectomy
Forbes/2005 [20]	1	M/75 yy	Left	200	Yes	N/A	CT	Adrenal cavernous hemangioma	Yes	Open adrenalectomy
Meng Ng/2008 [12]	1	M/59 yy	Left	31	Yes	Yes	CT	Adrenal cavernous hemangioma	No	Laparoscopic adrenalectomy
Nigri/2008 [53]	1	F/58 yy	Right	57	No	No	CT	Adrenal cavernous hemangioma	No	Laparoscopic adrenalectomy
Arkadopoulos/2009 [54]	1	F/35 yy	Left	80	No	No	CT, MRI	Adrenal cavernous hemangioma	No	Open adrenalectomy
Telem/2009 [28]	1	F/42 yy	Left	120	Yes	No	CT, MRI	Adrenal cavernous hemangioma	No	Laparoscopic adrenalectomy
Daisuke/2009 [19]	1	M/51 yy	Left	45	No	No	CT, MRI	Adrenal cavernous hemangioma	No	Laparoscopic adrenalectomy
Matsuda/2009 [55]	1	M/51 yy	Right	170	Yes	No	US, CT	Adrenal cavernous hemangioma	Yes	Open adrenalectomy
Alhajri/2010 [56]	1	M/75 yy	Left	180	Yes	No	CT	Adrenal cavernous hemangioma	No	Open adrenalectomy
Aljabri/2011 [57]	1	F/19 yy	Right	73	No	No	US, CT, MRI	Adrenal hemangioma	No	Laparoscopic adrenalectomy
Ellis/2011 [9]	9	F = 6 M = 3 42 yy	Right = 6 Left = 3	55	Yes = 6 No = 3	Yes = 5 No = 4	CT = 9	Adrenal cystic lymphangioma	No	N/A
Cakir/2012 [58]	2	F = 0 M = 2 51 yy	Right = 1 Left = 1	50	Yes = 2 No = 0	No	US, CT	Adrenal cystic lymphangioma	No	Open adrenalectomy
Oishi/2012 [22]	1	F/75 yy	Left	50	No	Yes	CT	Adrenal cavernous hemangioma	No	Laparoscopic adrenalectomy
Akand/2013 [59]	1	F/44 yy	Left	95	No	No	US, MRI	Adrenal cystic lymphangioma	No	Open adrenalectomy
Galea/2013 [60]	1	F/84 yy	Left	130	Yes	No	CT	Adrenal cavernous hemangioma	No	Open adrenalectomy
Liu/2013 [61]	1	F/45 yy	Left	30	No	No	US, CT	Adrenal cystic lymphangioma	No	Laparoscopic adrenalectomy
Lorenzon/2013 [26]	1	M/77 yy	Bilateral	100	No	Yes	CT	Adrenal cavernous hemangioma	No	Open adrenalectomy
Quildrian/2013 [35]	1	F/62 yy	Left	130	No	No	N/A	Adrenal cavernous hemangioma	No	Open adrenalectomy
Secil/2013 [62]	1	F/42 yy	Left	N/A	Yes	No	MRI	Adrenal cystic lymphangioma	No	Laparoscopic adrenalectomy
Aoyama/2014 [63]	1	F/47 yy	Left	N/A	Yes	No	CT, MRI	Adrenal lymphangioma	No	Open adrenalectomy
Blanchard/2014 [64]	1	F/23 yy	Right	52	Yes	No	CT	Adrenal cystic lymphangioma	No	Laparoscopic adrenalectomy
Edwards/2014 [65]	1	F/78 yy	Right	58	Yes	Yes	CT	Adrenal cavernous hemangioma	No	Laparoscopic adrenalectomy
Jung/2014 [14]	1	F/79 yy	Left	160	Yes	No	CT, MRI	Andrenal cystic lymphangioma	No	Laparoscopic adrenalectomy
Noh/2014 [23]	1	F/27 yy	Right	78	Yes	No	CT	Adrenal cavernous hemangioma	No	Laparoscopic adrenalectomy
Wang/2014 [66]	1	F/37 yy	Right	60	No	No	CT	Adrenal cavernous hemangioma	No	Laparoscopic adrenalectomy
Zhao/2014 [4]	3	F = 0 M = 3 50 yy	Right = 0 Left = 3	31	Yes = 0 No = 3	Yes = 0 No = 3	CT = 2 N/A = 1	Andrenal cystic lymphangioma = 3	No=3	Laparoscopic adrenalectomy=1 Open adrenalectomy=2
Agrusa/2015 [36]	1	F/49 yy	Right	110	Yes	No	CT	Adrenal cavernous hemangioma	No	Laparoscopic adrenalectomy
Bosnalı/2015 [67]	1	M/5 yy	Right	40	Yes	No	MRI	Andrenal cystic lymphangioma	No	Laparoscopic adrenalectomy
Gao/2015 [68]	8	F = 5 M = 3 46 yy	Right = 4 Left = 4	40	Yes = 4 No = 4	Yes = 6 No = 2	CT = 8	Adrenal cystic lymphangioma	No	Laparoscopic adrenalectomy=7 Open adrenalectomy=1
Geramizadeh/2015 [69]	1	F/43 yy	Left	25	Yes	No	CT	Adrenal cystic lymphangioma	No	Open adrenalectomy
Ho/2015 [70]	1	F/44 Y	Left	60	No	No	CT	Adrenal cystic lymphangioma	No	Laparoscopic adrenalectomy
Hodish/2015 [24]	1	M/59 yy	Left	22	Yes	Yes	CT, MRI	Adrenal cystic lymphangioma	No	Laparoscopic adrenalectomy
Joliat/2015 [71]	1	F/38 yy	Left	72	Yes	No	US, CT, MRI	Adrenal cystic lymphangioma	No	Open adrenalectomy
Lykoudis/2015 [72]	5	F = 3 M = 2 52 yy	Right = 1 Left = 4	97	Yes = 2 No = 3	Yes = 0 No = 5	CT = 5	Adrenal cavernous hemangioma=4 Adrenal cystic lymphangioma=1	No	Open adrenalectomy=5
Michalopoulos/2015 [73]	1	F/39 yy	Right	90	No	No	CT	Adrenal cystic lymphangioma	No	Open adrenalectomy
Pang/2015 [33]	1	F/71 yy	Left	94	Yes	No	CT	Adrenal cavernous hemangioma	No	Laparoscopic adrenalectomy
Tarchouli/2015 [31]	1	F/71 yy	Right	350	Yes	No	CT	Adrenal hemangioma	No	Open adrenalectomy
Nasir/2016 [74]	1	M/ N/A	Left	55	No	No	CT	Adrenal cystic lymphangioma	No	Open adrenalectomy
Nursal/2016 [18]	1	F/48 yy	Left	120	Yes	No	CT	Adrenal cavernous hemangioma	Yes	Open adrenalectomy
Rowe/2016 [30]	7	F = 4 M = 3 47 yy	Right = 5 Left = 2	43	Yes = 0 No = 7	Yes = 0 No = 7	CT = 7	Adrenal cystic lymphangioma	No	N/A
Yoshiaki/2016 [75]	1	M/77 yy	Left	54	No	No	CT	Adrenal cavernous hemangioma	No	Laparoscopic adrenalectomy
Feo/2018 [76]	1	M/70 yy	Left	83	No	No	CT, MRI	Adrenal cavernous hemangioma	No	Open adrenalectomy
Hashimoto/2018 [77]	1	M/70 yy	Left	158	Yes	Yes	CT	Adrenal cavernous hemangioma	No	Open adrenalectomy
Liechti/2018 [78]	1	F/32 yy	Left	125	Yes	No	CT, MRI	Adrenal cystic lymphangioma	No	Open adrenalectomy
Zheng/2018 [79]	25	F = 19 M = 6 50 yy	Right = 17 Left = 8	42	Yes = 11 No = 14	Yes = 3 No = 22	N/A	Adrenal cavernous hemangioma=17 Adrenal cystic lymphangioma=8	N/A	N/A
Bibi/2019 [80]	1	M/78 yy	Left	120	Yes	No	CT	Adrenal cystic lymphangioma	No	Open adrenalectomy
Degheili/2019 [81]	1	M/53 yy	Right	40	Yes	Yes	CT, MRI	Adrenal cystic lymphangioma	No	Open adrenalectomy
Degheili/2019 [82]	1	M/83 yy	Right	80	Yes	No	CT	Adrenal cavernous hemangioma	No	Open adrenalectomy
Nishtala/2019 [32]	5	F = 3 M = 2 64 yy	Right = 3 Left = 2	76	Yes = 3 No = 2	Yes=1 No=4	CT = 5	Adrenal cavernous hemangioma	No	Laparoscopic adrenalectomy
Koperski/2019 [83]	37	F = 26 M = 11 35 yy	Right = 21 Left = 16	45	Yes = 15 No = 22	Yes = 4 No = 33	N/A	Adrenal cystic lymphangioma	N/A	N/A
Zaghbib/2019 [84]	1	F/37 yy	Right	47	No	No	CT	Adrenal cystic lymphangioma	No	Laparoscopic adrenalectomy
Gupta/2020 [85]	1	M/ 79 yy	Right	69	Yes	No	CT	Adrenal cavernous hemangioma	No	Open adrenalectomy
Yaegashi/2020 [23]	1	F/33 yy	Left	70	Yes	No	CT	Adrenal cystic lymphangioma	No	Laparoscopic adrenalectomy
Al-Rawashdah/2021 [86]	1	M/58 yy	Left	70	Yes	No	CT	Adrenal cavernous hemangioma	No	Laparoscopic adrenalectomy
Huang/2021 [87]	1	M/67 yy	Right	95	Yes	No	CT	Adrenal cavernous hemangioma	No	Laparoscopic adrenalectomy
Issam/2021 [88]	1	M/46 yy	Right	35	Yes	No	CT	Adrenal cystic lymphangioma	No	Laparoscopic adrenalectomy
Kafadar/2021 [89]	1	F/39 yy	Right	86	Yes	No	CT, MRI	Adrenal cystic lymphangioma	No	Laparoscopic adrenalectomy
Wan/2021 [90]	1	M/68 yy	Left	28	No	No	CT, MRI	Adrenal cystic lymphangioma	No	Open adrenalectomy
Marques-Piubelli/2022 [3]	2	F = 2 M = 0 35 yy	N/A	120	Yes = 1 No = 1	No	CT = 2	Adrenal cystic lymphangioma	No	N/A
Our cohort	21	F = 9 M = 12 53 yy	Right = 9 Left = 12	47	Yes = 4 No = 11	Yes=4 No=11	CT = 15 N/A = 6	Adrenal cavernous hemangioma=12 Adrenal cystic lymphangioma=8 Adrenal cystic tumor=1	No	Laparoscopic adrenalectomy=20 Open adrenalectomy=1

*F* females, *M* males, *US* Ultrasound, *CT* Computerized Tomography, *MRI* Magnetic Resonance Imaging, *N/A* not available.

## Discussion

ACHs and ACLs are benign vascular neoplasms consisting of many entangled thin-walled and aberrant dilated vessels that are prone to rupture [9]. The first case of adrenal cavernous hemangioma (ACH) was reported in 1955 by Johnson and Jeppesen, while adrenal cystic lymphangioma (ACL) was first described in 1965 by Linn [10]. ACHs and ACLs usually arise from the adrenal cortex, as only two cases of adrenal medulla involvement have been described [11, 12]. All of our cases originated in the adrenal cortex, with only one case associated with medullary hyperplasia [5]. Dilated spaces delimitated by a single endothelial layer are typical histopathological features of ACHs and ACLs. ACHs are usually associated with necrotic and hemorrhagic areas separated by fibrotic septa. Phleboliths might be also present within the context of sinusoidal dilatation [13]. ACLs are characterized by unilocular or multilocular architecture with acellular, homogeneous, proteinaceous fluid within cystic spaces. Before the WHO 2022 guidelines, the presence of these histopathological features was sufficient to confirm the diagnosis, as observed in ten cases from our cohort. Immunohistochemical analysis was usually conducted in cases with ambiguous morphology. Typical immunohistochemical markers of ACHs are CD34 and CD31, whereas podoplanin is specifically expressed by lymphatic cells [14]. The literature data is consistent with the immunohistochemical findings in our cohort. Adrenal vascular tumors are typically benign, with only one case of malignant hemangioendothelioma described so far [15]. None of the cases in our cohort demonstrated malignancy on pathological analysis. The specific etiology remains unknown. ACHs could represent an expression of persistent pathological angiogenesis in the context of hereditary angiogenetic disorders [16]. ACLs are embryologically derived from malformed lymphatic vessels, although the blockage or inflammation of proximal lymphatics might be alternative causes [3, 9]. Diagnosis of adrenal vascular tumors is frequently made between the fifth and sixth decade of life, with a higher incidence in female subjects [3]. In our cohort, the median age (58 years; range 30–75 years) aligned with literature data. Higher incidence in female subjects is probably related to sex-hormonal influences, as estrogens might promote the development of hemangiomas in other body districts, such as in the liver or skin [17]. However, our cohort showed no sex differences, likely due to the limited sample size. ACHs and ACLs are usually discovered as adrenal incidentalomas, representing rare findings among adrenal masses. In our cohort, 67% of the cases were incidentally discovered, while about half of the cases reported in the literature typically presented with symptoms related to mass effect. This slight discrepancy may be due to the fact that most adrenal vascular cysts described in the literature are case reports, which often involve atypical or more symptomatic presentations. Although adrenal vascular cysts are generally asymptomatic, dull abdominal pain could appear when tumor diameter significantly increases, determining mechanical pressure on other structures in the retroperitoneal space. In our cohort, lumbar pain was reported in four patients. In addition, external insults might cause the rupture of the aberrant endothelial components of these tumors, thus resulting in a life-threatening retroperitoneal hemorrhage. The latter represents an extremely rare occurrence, as only 3 cases were described [18–20]. None of the cases in our cohort developed this complication. Symptoms related to hormonal hypersecretion are very uncommon, with most ACHs and ACLs resulting in non-functioning adrenal masses, as confirmed by our study. However, few cases of hypercortisolism, hyperaldosteronism, and catecholaminergic hypersecretion were reported [21–24]. Hormonal abnormalities were also observed in our cohort, including two patients with MACS and two with elevated urinary metanephrines. Because ACHs and ACLs do not usually show endocrine activity, these findings could be likely explained by the gathering of active metabolites of adrenal hormones due to an impaired vascular flow within tumor tissues [25]. Nevertheless, both DST and urinary metanephrine measurements may yield false positive results due to physiological factors, laboratory techniques, and/or interfering substances. All cases in our cohort were unilateral. This result is in line with literature data, with only 2 cases of bilaterality reported so far [26, 27]. Mass enlargement has frequently been reported, likely due to vascular or lymphatic ectasia, but most studies lack sufficient follow-up and detailed measurements of tumor diameter to assess an increasing trend in tumor size [28]. We found in 6 patients of our cohort a significant increasing tumor size over a mean follow-up period of 4 years. CT and MRI represent the diagnostic tool of choice. On CT scans, ACHs are heterogeneous and well-defined masses with areas of central necrosis and speckled calcifications, which are present in up to 87% of the cases [29]. Calcifications might be detected in ACLs as well [30]. In our cohort, only 24% of the cases presented with calcifications on CT imaging. This finding is probably due to the limited number of cases. Further enhanced CT features, such as heterogeneous pattern enhancement with progressive centripetal filling, can be useful for the assessment of ACHs [31]. ACLs are multilocular well-circumscribed lesions characterized by low internal attenuation density values with thin enhanced walls. Notably, we found in our cohort CT density values greater than 20 UH in the non-contrast series, which is considered a risk feature for malignancy according to the European Guidelines for adrenal incidentalomas [8]. This finding represents the main misleading characteristics in the diagnostic evaluation of these tumors, as high attenuation values without contrast washout on CT scans can be found also in malignant tumors [32]. The presence of additional imaging features, such as large size, rapid growth, irregular shape, calcifications, and necrosis, may complicate the diagnostic assessment. On MRI, ACHs usually appear as non-homogeneous hypointense masses with central hyperintensity on T1-weighted images and peripheral hyperintensity on T2-weighted images due to necrosis, hemorrhagic areas, and multiple vascular cavities [13, 33]. Peripheral nodules are also representative of cavernous sinuses [34]. MRI characteristics of ACLs are low signal intensity on T1-weighted images and high intensity on T2-weighted images. We chose not to analyze MRI characteristics in our cohort, as MRI acquisitions lack quantitative parameters with specific physical meaning. Treatment guidelines for ACHs and ACLs have not been established yet due to their rarity. The treatment usually differs according to tumor size, pre-operative imaging characteristics, and functional status. Surgery is indicated for functioning adrenal masses and/or larger than 6 cm because of the higher risk of malignancy and retroperitoneal hemorrhage [35]. On the other hand, tumors with cystic appearance smaller than 4 cm are likely to be benign, thus a conservative approach with active surveillance based on endocrinological and radiological assessments can be considered. For adrenal masses measuring 4–6 cm, patient’s preference and clinical conditions should be considered. As the risk of hemorrhage increases with tumor size, surgical removal of these lesions remains the unique option to prevent life-threatening retroperitoneal hemorrhage. Nowadays laparoscopic adrenalectomy represents the procedure of choice for both ACHs and ACLs as the risk of rupture after surgical manipulation is relatively low because of the fibrotic capsule surrounding the vascular tumor [25]. All of our cases underwent laparoscopic adrenalectomy, with only one case of conversion due to intraoperative bleeding. However, most ACHs and ACLs described in the literature usually underwent open adrenalectomy due to their size and misleading imaging features, especially in the past [36].

## Conclusions

ACHs and ACLs have been detected with increased frequency due to the improvement of imaging techniques. However, they represent a diagnostic dilemma in clinical practice due to their rarity and their misleading imaging features overlapping with adrenal malignant tumors. Because of the heterogeneous clinical and radiological pictures, treatment should be targeted to the patient’s characteristics. Therefore, if an adrenal vascular tumor is suspected, treatment options must be discussed by a multidisciplinary team including endocrinologists, radiologists, pathologists, and surgeons, with expertise in the differential diagnosis of adrenal tumors.

## Data Availability

Data is provided within the manuscript
